# THE ASSOCIATION BETWEEN SUBJECTIVE AGE AND TECHNOLOGY USE AMONG OLDER ADULTS

**DOI:** 10.1093/geroni/igac059.2189

**Published:** 2022-12-20

**Authors:** Tomoko Ikeuchi, Tomoko Wakui, Sakiko Itoh, Hiroyasu Miwa, Kentaro Watanabe

**Affiliations:** Tokyo Metropolitan Institute of Gerontology, Tokyo, Tokyo, Japan; Tokyo Metropolitan Institute of Gerontology, Tokyo, Tokyo, Japan; Osaka University, Suita, Osaka, Japan; National Institute of Advanced Industrial Science and Technology, Kashiwa, Chiba, Japan; National Institute of Advanced Industrial Science and Technology, Kashiwa, Chiba, Japan

## Abstract

**Background:**

Subjective age (SA) (i.e., felt age) has been found to be a biopsychosocial marker of aging. This study examined the associations between SA and frequency of technology usage of older adults.

**Methods:**

Data were collected via an online survey conducted in 2020. The study analyzed participants aged 65 to 89 (M = 71.9, SD = 3.91) years resided in Japan (N = 1855, 54.3% women). SA was indexed by asking participants to specify in years how old they felt. Proportional discrepancy scores (PDS) ((SA - chronological age) / chronological age) were calculated to indicate younger or older SAs and used as an independent variable. Participants were asked about the frequency of computer, smartphone, flip phone, and SNS use.

**Results:**

Nearly 90% reported using computers for more than 2-3 days a week, 64.3% smartphones, 22.9% flip phones, and 36.6% SNS. Logistic regression analyses revealed that lower PDS (i.e., feeling younger) was associated with a significantly higher frequency of smartphone use (OR: 0.75; 95% CI: 0.59, 0.96) after adjusting for age, gender, education, and subjective health. No such association was found for computer, flip phone, and SNS use. Implications: Older adults who use smartphones daily may feel younger than those who do not. Since the present study was administered during the COVID-19 pandemic, the daily use of smartphones may have helped older adults stay in touch with friends and family members and obtain information they need. The use of smartphones possibly contributed to better mental health outcomes while practicing social distancing.

